# Optimization of Anchovy–Threadfin Bream Composite Surimi: I-Optimal Mixture Design for Sensory Enhancement and Impact Assessment of Three Exogenous Proteins

**DOI:** 10.3390/foods15081417

**Published:** 2026-04-17

**Authors:** Xiayin Ma, Shihao Chen, Jingfu Bai, Shixian Yin, Zhixing Rong, Hu Hou, Wenli Kang

**Affiliations:** 1 State Key Laboratory of Marine Food Processing & Safety Control, College of Food Science and Engineering, Ocean University of China, No. 1299, Sansha Road, Qingdao 266404, China; maxiayin@csust.edu.cn; 2 School of Food Science and Bioengineering, Changsha University of Science and Technology, 960, 2nd Section, Wanjiali South RD, Changsha 410114, China; 18274839886@163.com (S.C.); m17872366470@163.com (J.B.); 3 Pingjiang Jinzai Food Co., Ltd., Yueyang 414517, China; yinshixian@jinzaifood.com.cn (S.Y.); rongzhixing@jinzaifood.com.cn (Z.R.)

**Keywords:** anchovy, composite surimi, mixture design, gel properties, exogenous proteins

## Abstract

The anchovy (*Engraulis japonicus*) is a highly abundant but underutilized fish resource in China, primarily due to its extreme post-harvest perishability. This study expanded the utilization of anchovy by developing a blended surimi from anchovy and golden threadfin bream, an I-optimal mixing design experiment was performed to optimize the formulation, and the effects of soy protein isolate (SPI), egg white powder (EWP), and yeast protein (YP) on the gel properties were investigated. The results of sensory evaluation and model prediction indicated that SPI had the most pronounced positive effect on the sensory characteristics of the gels, especially improving the elasticity, followed by EWP. Furthermore, the SPI-rich sample exhibited superior gel strength and chewiness, which was attributed to the increased β-sheet structure and the highest content of disulfide bonds in the protein network. And the water hold capacity of SPI-rich sample increased by 6.0%. The YP-rich group showed the strongest hydrophobic interactions and exhibited a significant enhancement in water hold capacity of 7.7%, which also provided a notable improvement in gel strength. The results showed that EWP contributed to the smoothness of the surimi, but it had no significant impact on water distribution, water-holding capacity, or the content of disulfide bonds within the gel network. Moreover, the EWP-rich group exhibited reduced the gel strength, hardness, and chewiness of the gel, resulting in the lowest overall sensory score of the surimi. Therefore, the optimal composite ratio was determined to be SPI:EWP:YP = 5.45%:2.55%:2.00%. These findings provided a precise blending strategy for developing high-quality surimi products from anchovy, offering a viable technical pathway for the value-added utilization of this resource.

## 1. Introduction

The Japanese anchovy (*Engraulis japonicus*) is a highly abundant yet underutilized fish resource in the Northwest Pacific, especially in China’s coastal waters [1]. In countries such as China, Japan, and South Korea, anchovies are consumed as an important dietary source of calcium and protein. They are rich in polyunsaturated fatty acids (PUFAs), notably eicosapentaenoic acid (EPA) and docosahexaenoic acid (DHA) [2]. Additionally, anchovies contain various flavor-enhancing compounds, including vitamins (A, B1, B2, niacin, and B-complex), minerals, free amino acids, nucleic acid-related substances, and betaine [3]. Given this nutrient profile, anchovies represent a valuable fishing resource and a raw material suitable for producing nutritious processed foods.

The efficient utilization of anchovies is severely constrained by their extreme perishability post-harvest, a phenomenon known as “water-induced decay.” Therefore, anchovies are mostly sun-dried, while a small number are pickled and fermented for consumption in coastal areas. Currently, anchovy processing primarily focused on the production of fish meal or the extraction of fish oil [4] and small-scale canned anchovy products have also appeared on the market. However, the potential of anchovies has not yet been fully realized, and appropriate processing methods could expand their range of applications. Given the potential for developing new products based on this species, developing surimi from anchovy alone is considered as a viable approach to counter its perishability. However, the inherent limitations of anchovy myofibrillar proteins often result in relatively weak gel strength and poor water-holding capacity, which is a challenge for producing high-quality surimi gel. To overcome this, the strategy of formulating composite surimi is commonly employed to enhance the gel-forming ability of the product.

A series of studies have demonstrated that blending anchovy with other fish species can effectively improve the texture, flavor, and economic efficiency of surimi products. Specifically, research on the anchovy and silver carp surimi blending system identified a 1:9 ratio as optimal, which maximized gel strength, thermal stability, and storage modulus while forming a dense and uniform network structure. The improvement was attributed to promoted myosin heavy chain cross-linking and aggregation, driven by favorable protein conformational changes such as decreased α-helix and increased β-sheet content during heating [5]. Moreover, this ratio also enhanced the overall flavor acceptability by beneficially altering the relative composition of key flavor compounds [6]. In a different study, Maza-Ramírez et al. added jumbo squid arm surimi at a 7:3 ratio to anchovy surimi to improve its texture and color. This resulted in a significant increase in hardness, elasticity, gel strength, and whiteness because the proteins from the two sources exhibited complementary functional properties [7].

In the processing of surimi products, adding exogenous proteins is a common strategy to improve their functional properties. A study investigated the effects of adding soy protein isolate (SPI) to silver carp surimi under various setting conditions, including temperature, time, and protein concentration. The results showed that the effect of SPI was highly dependent on the heat treatment, which was explained by the possibility that SPI could lower surimi protein concentration or inhibit the protease activity that causes “modori” (gel deterioration), protecting the gel network at higher temperatures [8]. Another study systematically investigated the effects of adding egg white protein (EWP) to squid surimi. The experimental results showed that when the ratio of surimi to EWP was 16:1, the best gel hardness, elasticity, breaking strength, cooking yield, and water-holding capacity could be achieved. The mechanism of action was attributed to EWP acting as a “gel scaffold”, which could regulate the kinetics of protein thermal aggregation, thereby promoting more complete protein unfolding and structural rearrangement and forming a more uniform gel network structure [9]. In addition, research has also explored how the addition of yeast β-glucan at different levels (0–5%) affects the quality of silver carp surimi gel. The study showed that adding about 2% yeast β-glucan provided the optimal benefits: significantly improved gel properties, reduced fishy odor, and enhanced sensory acceptance of the product [10].

In this study, anchovy surimi was blended with surimi from golden threadfin bream (*Nemipterus virgatus*), a conventional high-quality raw material valued for its superior gelling properties, neutral flavor, and white color. This combination exploits their complementary functionalities, with threadfin bream providing a robust elastic gel network and anchovy contributing nutritional and distinct flavor characteristics. Prior research supports the synergistic potential of mixed surimi systems, demonstrating that composite gels can exhibit enhanced gel strength, water-holding capacity, whiteness, and a more compact network structure compared to single-species gels [11]. The incorporation of exogenous proteins-SPI, EWP, and yeast protein (YP)-offers a cost-effective strategy for further quality improvement. To systematically investigate the individual and interactive effects of these proteins on the composite surimi, a mixture design experiment was employed. The objective was to quantitatively determine the optimal protein combination for maximizing gel strength, water-holding capacity, and microstructural uniformity. This work presents a systematic analysis of ternary protein interactions within an anchovy–threadfin bream surimi matrix. A quantitative model for synergistic optimization was established, providing a practical and precise blending strategy to develop higher-value surimi products from anchovies, thereby enhancing the resource utilization and commercial potential of this species.

## 2. Materials and Methods

### 2.1. Materials

Anchovies were procured from China Aquatic Products Co., Ltd. (Beihai, China), with an average body length of 6 ± 1 cm after decapitation and evisceration. The processed fish were frozen at −20 °C for future use. Frozen golden thread fish surimi (AAA grade) was purchased from Jingli Aquatic Products Co., Ltd. (Honghu, China). YP was provided by Angel Yeast Co., Ltd. (Yichang, China). EWP was purchased from Runbu Biotechnology Co., Ltd. (Shijiazhuang, China). SPI was supplied by Yuncheng Biotechnology Co., Ltd. (Jinan, China). The complex phosphates used in this study was formulated with sodium tripolyphosphate, sodium pyrophosphate, and sodium hexametaphosphate at the ratio of 2:2:1. Food-grade additives, including cassava starch, sucrose, monosodium glutamate, and edible salt, were purchased from local supermarkets in Changsha, China.

### 2.2. Surimi Gel Preparation

Anchovies were decapitated, eviscerated, and rinsed with ice-cold water until surface impurities were removed. Surface moisture was eliminated using defatted gauze prior to processing. Frozen golden threadfish surimi was thawed to a cuttable state. Supplementary ingredients were added according to Formula 1 (Table 1). The prepared anchovies and threadfin surimi were ground in a meat grinder, with sequential incorporation of polyphosphates, edible salt, and seasonings. Ice-distilled water was gradually introduced to adjust the moisture content to 78–80% (*w*/*w*)

The homogenized fish mixture was molded into 1.8 × 1.8 × 1.8 cm^3^ silicone matrices. A two-stage heating protocol was implemented: primary gelation at 40 °C for 20 min followed by secondary cooking at 90 °C for 10 min. Post-heating, the fish surimi gel were immediately quenched in ice-water for 30 min and subsequently stored at 4 °C for 24 h to complete gel maturation. All processing steps were conducted under controlled temperature (4 °C ± 1 °C) and time conditions. Three independent batches were prepared on different days to account for biological variability.

### 2.3. Optimal Mixing Design Experiment

The sensory attributes of elasticity, cohesiveness, and smoothness were selected for evaluation based on preliminary tests, as they are recognized as key textural properties determining the overall acceptability of surimi gels. Preliminary experiments revealed that individual incorporation of three protein types (SPI, EWP, YP) at 2–6% concentrations exhibited a unimodal effect on textural properties, with initial improvement followed by deterioration. This range was selected to cover the typical effective dosage of exogenous proteins in surimi systems and to identify the optimal level before potential negative effects of over-addition. Consequently, an I-optimal mixture design was implemented via Design Expert 2022 software (Stat-Ease Inc., Minneapolis, MN, USA) to determine the optimal ternary blend ratio of SPI, EWP, and YP in anchovy surimi gel formulations. The sensory evaluation was conducted by 20 food professionals to assess elasticity, cohesiveness, and smoothness using a 50-point scale under controlled conditions.

Prior to formal evaluation, all panelists underwent training sessions using reference samples representing a wide spectrum of gel textures (e.g., high vs. low elasticity, cohesiveness, and smoothness). Consensus was reached on the definitions and scoring scales for each attribute (elasticity, cohesiveness, smoothness) to ensure scoring consistency. During training, panelists’ discrimination and reproducibility were validated using duplicate samples.

### 2.4. Preparation of Surimi Gels with Different Protein Compositions

Based on the formulation ratios in Table 1 and the gel preparation method in Section 2.2, the proportions of soy protein isolate, yeast protein, and egg white powder were determined, as shown in Table 2.

### 2.5. Determination of Gel Strength

The surimi gels (1.8 × 1.8 × 1.8 cm^3^) were equilibrated to ambient temperature (25 ± 1 °C), and their gel strength was measured using a TA.XT Express texture analyzer (Stable Micro Systems, Godalming, UK). A cylindrical P/50 aluminum probe (5 mm diameter) was used to compress the samples at a test speed of 50 mm/min with a trigger force of 0.75 N and penetration depth of 10 mm. Gel strength (g·cm) was calculated as the product of breaking force (g) and deformation distance (mm) at the first fracture point.

### 2.6. Textural Profile Analysis (TPA)

After the surimi gels were equilibrated to room temperature, their texture parameters, including hardness, elasticity, adhesiveness, cohesiveness, chewiness, and rebound, were measured using a texture analyzer (e.g., TA-XT plus or similar model) equipped with a metal cylindrical probe (P/36). The analysis was performed under the following conditions: a two-cycle compression test with a 5 s interval between compressions, 50% deformation, a pre-test speed of 3.0 mm/s, a test speed of 60 mm/min, a post-test speed of 3.0 mm/s, and a trigger force of 5.0 g.

### 2.7. Determination of Water-Holding Capacity

The water-holding capacity (WHC) was determined following the method of Pizarro-Oteíza [12] with modifications. Briefly, accurately weighed gel samples (2 g, recorded as *m*_1_) were placed between two layers of filter paper and centrifuged at 8000 rpm (7104× *g*) at 4 °C for 15 min using a CenLee16R centrifuge (Hunan Xiangli Scientific Instrument Co., Ltd., Changsha, China). After centrifugation, the filter paper was removed, and the samples were immediately reweighed (*m*_2_). The procedure was performed in triplicate for each sample group to ensure reproducibility. The WHC was calculated as follows:

$$\mathrm{WHC}\;(\%)\;=\;\frac{m_{2}}{m_{1}}\times 100$$

### 2.8. Dynamic Temperature Sweep

The rheological methods were applied as previously described, with some modifications [13]. The temperature-dependent rheological properties of fish surimi sol were analyzed using a Discovery HR 30 dynamic rheometer (TA Instruments Ltd., New Castle, DE, USA) with 40 mm parallel steel plates. A fixed gap of 1.5 mm was set between the plates. A temperature sweep test was performed under the following conditions: a fixed frequency of 1 Hz, constant stress of 10 Pa, and a heating rate of 2 °C/min from 25 °C to 90 °C, with temperature controlled by the instrument’s built-in Peltier system. Prior to the test, stress sweeps (0.1–500 Pa) were conducted at 25 °C, 40 °C, and 90 °C to confirm that all measurements remained within the linear viscoelastic region (LVR), ensuring the structural integrity of the sample during testing.

### 2.9. Low-Field Nuclear Magnetic Resonance (LF-NMR)

The water distribution characteristics of surimi gel were analyzed using a low-field nuclear magnetic resonance (LF-NMR) analyzer (NMI20-060H-I, Niumag Analytical Instruments Co., Suzhou, China). Surimi gel samples were placed in NMR glass tubes (2 cm diameter × 4 cm height) and subjected to T_2_ relaxation time measurements at 25 °C. The Carr–Purcell–Meiboom–Gill (CPMG) sequence was employed with the following parameters: 10,000 echoes (NECH), an 8000 ms wait time (TW), a 0.1 ms echo time (TE), and 16 scan repetitions. All relaxation measurements were conducted under temperature-controlled conditions (25 °C). Acquired relaxation data were processed using a T_2_-CPMG curve-fitting algorithm to determine T_2_ distributions, enabling quantitative characterization of water mobility and spatial heterogeneity within the gel matrix.

### 2.10. Magnetic Resonance Imaging (MRI)

Low-field nuclear magnetic resonance (LF-NMR) analysis was performed using an NMI20-060H-I analyzer (Suzhou Niumag Analytical Instruments Co., Suzhou, China) with optimized spin-echo pulse sequences. Acquired gray-scale images were processed through uniform intensity mapping followed by pseudo-color transformation in OsiriX 7.5.1 (Pixmeo, Bernex, Trimbach, Switzerland) to generate enhanced color-coded visualizations.

### 2.11. Chemical Forces Analysis

Chemical forces between surimi protein molecules were analyzed according to the reported method [14]. Five solutions were prepared to analyze the contributions of different chemical interactions: (1) 0.05 mol/L NaCl (SA, control for ionic bonds), (2) 0.6 mol/L NaCl (SB, baseline for hydrogen bonds), (3) 1.5 mol/L urea + 0.6 mol/L NaCl (SC, reference for hydrophobic interactions), (4) 8 mol/L urea + 0.6 mol/L NaCl (SD, baseline for disulfide bonds), and (5) 8 mol/L urea + 0.6 mol/L NaCl + 0.5 mol/L β-mercaptoethanol (SE, disrupt disulfide bonds). Fish surimi gel samples were immersed in each solution, homogenized for 3 min, and incubated at 4 °C for 1 h. After centrifugation at 7104× *g* for 15 min, the supernatant protein content was quantified. Chemical interaction strengths were calculated as follows: ionic bonds (Absorbance Ratio: SB-SA), hydrogen bonds (Absorbance Ratio: SC-SB), hydrophobic interactions (Absorbance Ratio: SD-SC), and disulfide bonds (Absorbance Ratio: SE-SD).

### 2.12. Fourier Transform Infrared Attenuated Total Reflection Spectroscopy (FTIR-ATR)

Freeze-dried fish surimi gels were analyzed by Fourier transform infrared (FT-IR) spectroscopy using a Nicolet Nexus 470 spectrophotometer (Thermo Scientific, Cheshire, UK) equipped with a universal attenuated total reflectance (ATR) accessory. Spectra were acquired in the mid-infrared region (1000–4000 cm^−1^) at 4 cm^−1^ resolution with 32 cumulative scans per sample to ensure optimal signal-to-noise ratio. Prior to measurement, background interference was eliminated by subtracting the spectrum of a clean ATR crystal. Acquired spectra were processed using OMNIC software (Thermo Scientific) for baseline correction and spectral smoothing, enabling precise identification of characteristic vibrational bands, including amide I (1600–1700 cm^−1^) and amide II (1500–1600 cm^−1^) for protein secondary structure analysis.

### 2.13. Scanning Electron Microscope (SEM)

The hydrogel microstructure was characterized using a field-emission scanning electron microscope (JSM-7900F, JEOL Ltd., Tokyo, Japan). Cubic gel specimens (1 × 1 × 1 mm^3^) were precisely sectioned with a surgical scalpel and chemically fixed in 2.5% glutaraldehyde at 4 °C for 24 h to preserve native ultrastructure. Following fixation, samples were subjected to freeze-drying to minimize dehydration-induced artifacts. Imaging was performed at an acceleration voltage of 3 kV under high vacuum conditions, optimizing secondary electron detection for high-resolution surface topography visualization.

### 2.14. Statistical Analysis

All experiments were performed in triplicate. Data were processed and plotted using SPSS 26.0 (SPSS Inc., Chicago, IL, USA) and Origin 2024 (Origin-Lab Corp., Northampton, MA, USA). Statistical significance at *p* < 0.05 was determined using the ANOVA function. For the mixture design experiment, model fitting, analysis of variance (ANOVA), and optimization were performed using Design Expert 2022 software. The adequacy of the models was evaluated using lack-of-fit tests and coefficients of determination (R^2^).

## 3. Results and Discussion

### 3.1. The Sensory Evaluation of Surimi Gels

**Figure 1:**
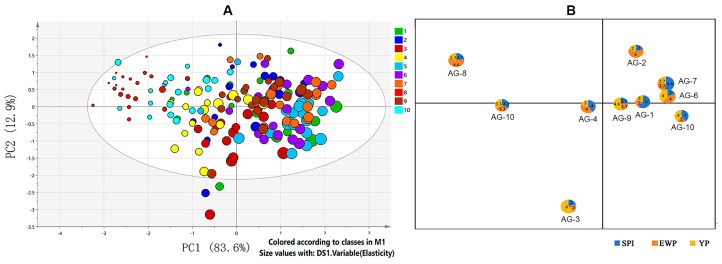
(**A**) Principal component analysis (PCA) score plot; (**B**) quadrant classification of samples based on principal components.

The mean liking scores for ten groups of composite surimi products are shown in Table 3. The sample AG-5 obtained the highest mean score, followed by samples AG-6, AG-7 and AG-1, respectively. AG-8 was the least preferred sample. The symmetric plot performed by principal component analysis (PCA) revealed distinct distribution patterns, with the first two principal components accounting for 96.5% of the total variance. The individual score points of each sensory panelist are presented in Figure 1A, while the centroids of each group are displayed in Figure 1B. Samples with a high proportion of SPI (AG-5, AG-7, AG-1) clustered together, as these samples all possessed high elasticity. Samples with a high proportion of EWP (AG-8 and AG-2) clustered on the positive axis of PC2, which was associated with higher smoothness. Samples with a high YP content showed no significant correlation with either principal component. These results indicated that SPI may contribute to the elastic texture of surimi, which consequently enhanced the consumer acceptability. In contrast, although EWP improved the smoothness of surimi gel, it significantly impaired the gel structure, resulting in reduced consumer acceptability. In addition, a balanced SPI-YP ratio is critical for optimizing the textural properties of anchovy–threadfin bream composite surimi gels.

### 3.2. Optimal Formula for Surimi Gel

Prediction models were developed to estimate three key sensory attributes (elasticity, toughness, and smoothness) and the overall total acceptability score. As summarized in Table 4, the analysis of variance (ANOVA) revealed that all F-tests for the models were statistically significant (*p* < 0.05), confirming that the ten formulations exhibited significant differences in terms of elasticity, toughness, smoothness, and total acceptability score. Furthermore, significant interactions were observed between SPI, EWP, and YP (*p* < 0.05), highlighting the complex synergistic effects within this blended protein system. The high R-values^2^ of the prediction models demonstrated a strong goodness of fit, as these models effectively capture the relationships between ingredient proportions and sensory responses, thereby providing a reliable framework for optimizing sensory properties based on compositional variations. The adequacy of the models was evaluated using the lack-of-fit test. As shown in Table 4, for the prediction model of the overall sensory score (Total), the *p*-value of the lack-of-fit test was 0.0633 (>0.05), indicating that the lack of fit was not significant at the 95% confidence level. This confirms that the selected model is adequate and fits the experimental data well. Furthermore, the coefficients of determination (R^2^) for all models were above 0.9271, demonstrating that the models possess satisfactory explanatory power.

**Figure 2:**
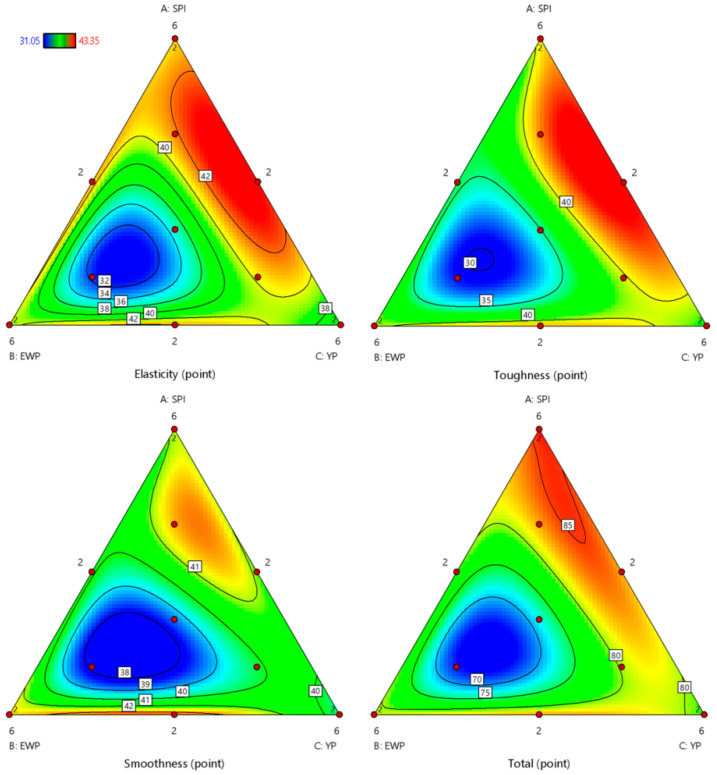
Contour plots of elasticity, toughness, smoothness, and total score for soy protein, yeast protein, and egg white powder. The red points in the figure indicate the experimental design points.

As shown in Figure 2, the contour plots illustrated the effects of three proteins on the sensory attributes (elasticity, toughness, smoothness) and the overall acceptability score. A color gradient from deep red (the highest response level) to deep blue (the lowest response level) effectively illustrated the variation in overall sensory acceptability across different combinations of the independent variables. The blue regions were predominantly localized in the EWP-dominated systems across all four plots. Conversely, the red regions were primarily associated with SPI-dominated systems and exhibited a tendency to extend towards areas influenced by YP. Based on the contour plots (Figure 2) and the response value prediction equation (Table 2), the proposed optimal ratio was identified as SPI:EWP:YP = 5.45:2.55:2. This distribution pattern reveals that SPI exerted the most significant contribution to the evaluated sensory metrics, followed by YP, whereas EWP had the least impact. Nevertheless, it is noteworthy that specific combination ratios of EWP and YP produced synergistic effects, which contribute positively to certain attributes, particularly smoothness. In addition, the interaction terms between SPI and YP consistently had the smallest *p*-values among all pairwise interactions, as summarized in Table 5, This indicated that the interaction between these two components exerted the most significant effect across the measured responses within the composite system. Furthermore, among all pairwise protein interactions, those involving SPI were consistently associated with the smallest *p*-values, further underscoring its dominant role and profound influence within the system.

### 3.3. Gel Strength and Textural Profile Analysis (TPA)

**Figure 3:**
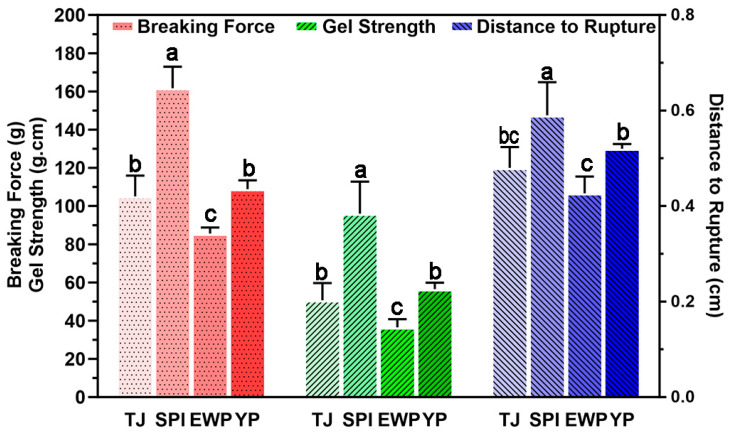
Effects of different dominant proteins on the breaking force, breaking distance, and gel strength of surimi gels. The control group (hereafter referred to as TJ, representing the anchovy (T)-threadfin bream (J) composite surimi). The lowercase letters (a–c) indicate statistically significant differences.

To further investigate the effects of three exogenous proteins on the textural quality of surimi, gel samples were prepared with three different protein blends, which were characterized by distinct main protein sources (SPI, YP, or EWP), and subjected to gel strength and TPA measurement. As illustrated in Figure 3, the SPI-rich group exhibited a significant enhancement in the breaking force, gel strength, and the distance to rupture compared with the control group (TJ). The gel strength of the SPI-rich group reached a maximum value of 92 g·cm. In contrast, the gel strength of the EWP-rich group decreased significantly, reaching only 38 g·cm. The YP-rich group, however, displayed no statistically significant alterations compared to the control group. The textural profiling revealed distinct differences among three protein-fortified samples. As shown in Table 6, the SPI-rich group exhibited the highest values for hardness (3551.76 ± 185.83 g), chewiness (2400.73 ± 223.22 g), and cohesiveness (0.45 ± 0.02). The YP-rich group exhibited moderate but notable increases in hardness (2948.46 ± 235.47 g) and chewiness (1655.46 ± 200.03 g) compared to the control group. Conversely, the EWP-rich group was characterized by a substantial reduction in both hardness (1792.6 ± 203.71 g) and chewiness (515.64 ± 90.57 g) compared to the control group. In summary, the texture and mechanical properties of surimi gel are significantly regulated by the type of exogenous protein.

It has been reported that SPI not only enhanced the gel strength of surimi through physical filling in its undenatured state but also promoted the formation of disulfide bonds and hydrophobic interactions, thereby reinforcing the three-dimensional gel network [15]. Similarly, Xu [16] demonstrated that the incorporation of YP promoted myosin-myosin cross-linking, resulting in a denser and more uniform microstructure with reduced void spaces, which consequently strengthened the gel matrix. In contrast, Wasinnitiwong [17] found through SDS-PAGE and TCA-soluble peptide analysis that EWP combined with κ-carrageenan did not influence myofibrillar cross-linking. These findings indicated that EWP primarily acted as a physical filler to improve gel strength, but excessive addition impaired the quality of surimi gels. Generally, the functional properties of these exogenous proteins are key determinants of gel enhancement in surimi systems. Compared to YP, SPI exhibited a higher density of charged groups and greater solubility [18]. These suggested that SPI had superior dispersibility within the surimi matrix and a stronger propensity to interact with charged residues on myosin, thereby more effectively facilitating the formation of the gel network.

### 3.4. Rheological Properties

**Figure 4:**
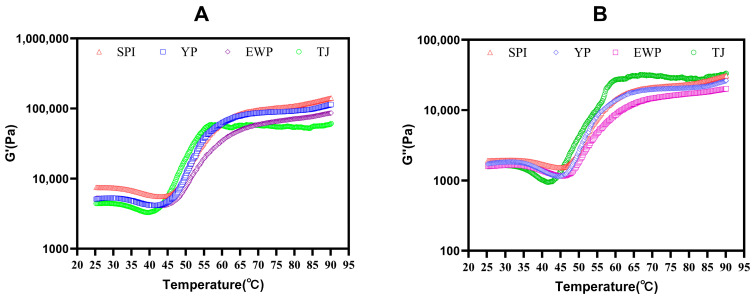
Characterization of surimi gels with different dominant proteins: (**A**) storage modulus and (**B**) loss modulus; the curves shown are representative of three independent measurements.

Dynamic temperature sweep rheology is widely utilized to evaluate the viscoelastic properties of surimi gels by monitoring the characteristic parameters of storage modulus (G′) and loss modulus (G″) during heating [19]. The dynamic rheological curves for G′ and G″ are presented in Figure 4A,B, respectively. All samples exhibited typical thermosetting gel behavior, as G′ was consistently higher than G″ throughout the heating process from 25 °C to 90 °C, confirming their predominant elastic solid character [20]. And pronounced differences in rheological behavior were observed among samples. The control group exhibited the lowest G′ and the highest G″ values, with the smallest gap between the two moduli. This pattern indicated that the sample of the control group had a typical viscoelastic character with insufficient gel hardness, reflecting a relatively weak and less rigid network structure. Compared to the control group, the gels supplemented with exogenous proteins showed significantly higher G′ than G″, in which the SPI-rich group exhibited the most favorable rheological properties, followed by the YP-rich group. While the EWP-rich group showed the poorest performance. The underlying mechanisms for these differences may be related to the distinct thermal behaviors of each exogenous protein and their interactions with the myosin network. Myosin, the primary gelling protein in surimi, forms a thermoreversible gel framework dominated by non-covalent interactions at 55–70 °C. And the thermal denaturation temperature of major SPI components (7S and 11S globulins) is 70–90 °C, which closely coincides with the high-temperature stabilization phase of the surimi gel. This temperature-dependent sequence indicates that SPI undergoes large-scale aggregation only after the initial myosin network has been established, which could fill and reinforce the pre-formed structure rather than interfering with its formation [21]. In comparison, YP contained a complex mixture of proteins with a broad molecular weight distribution, which lacked a distinct, uniform, and strongly self-assembling capacity [22]. Moreover, the lower content of sulfur-containing amino acids (cysteine/cystine) in YP limited its capacity to build high-strength networks via disulfide cross-linking [23]. The inferior rheological properties observed in the EWP-rich gel may be attributed to the denaturation temperature (56–65 °C) of ovalbumin in EWP. This temperature is directly overlapped with the critical temperature window (45–65 °C) for the formation of the myosin gel network [24]. Therefore, preformed elliptical protein soft aggregates can only enhance the gel strength of surimi through physical filling, with limited effectiveness [25].

### 3.5. Water Holding Capacity of Surimi Gels

**Figure 5:**
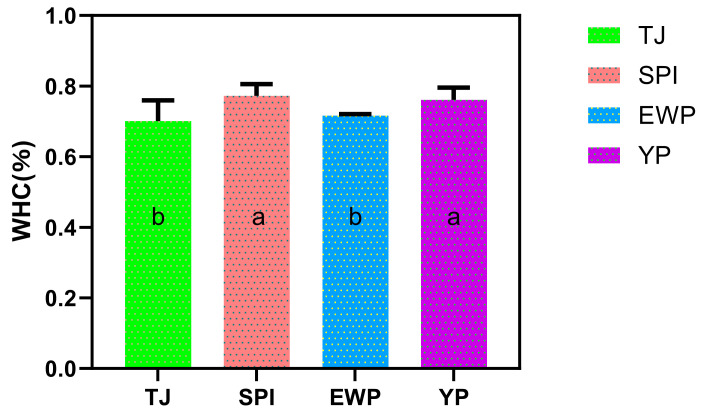
Effects of different dominant proteins on the WHC of surimi gels. Different letters (a,b) indicate significant differences.

The WHC of surimi gels incorporated with different exogenous proteins was presented in Figure 5. Compared to the control group (TJ), gels of the SPI-rich and YP-rich groups exhibited a significant enhancement in WHC; they increased by 7.7% and 6.0%, respectively. The WHC of gels was influenced by complex factors related to protein composition and interactions. It has been reported that SPI contained numerous hydrophilic molecule groups, and their inherent gel network exhibited high WHC [26]. When incorporated into surimi, SPI introduced these water-retentive structures into the composite system, resulting in a significant enhancement in the water-holding capacity of the surimi gel. In addition, the nearly neutral pH of SPI could keep the system away from the isoelectric point of myofibrillar proteins, which increased the net charge and electrostatic repulsion among proteins, enhancing their hydration and WHC [27]. YP may contribute to WHC through a different mechanism, as yeast protein commonly contains a certain amount of cell-wall-derived β-glucans [28]. It has been reported that β-glucans could function as hydrophilic colloids, directly binding a substantial amount of water. Their inherent viscosity modifies the system’s rheology, effectively reducing syneresis and moisture loss during thermal processing [29]. The EWP-rich group showed no significant improvement in WHC. This could be explained by the mismatch in reaction kinetics between EWP gelation and myofibrillar protein network formation; the thermal gelation of ovalbumin in EWP occurs around 56–65 °C, which directly overlaps with the critical temperature window (45–65 °C) for myosin network formation [30]. This simultaneity may limit EWP’s effective integration into the covalent network of fish proteins, restricting its contribution to a reinforcing filler role with less impact on the overall network’s water-binding capacity.

### 3.6. LF-NMR and Thermal Imaging

**Figure 6:**
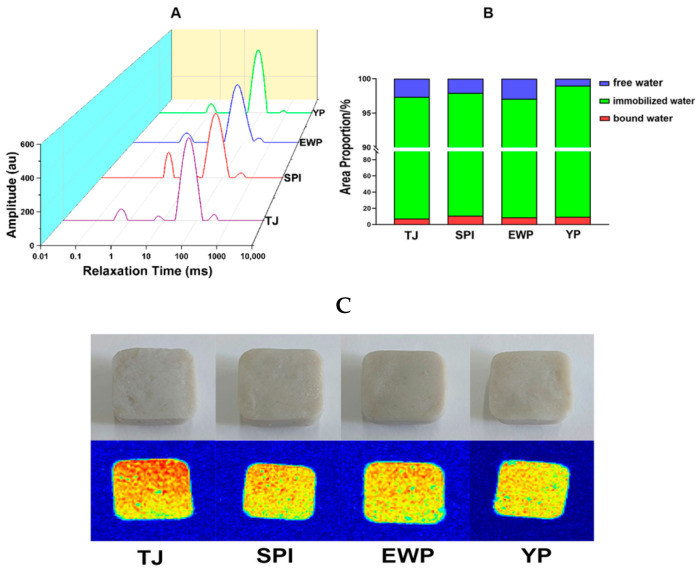
T_2_ relaxation time (**A**), area proportion of various water peaks (**B**), and physical image and NMR spectrum (**C**) of surimi gels with different dominant proteins; the spectra and images shown are representative of three independent experiments.

The relaxation time (T_2_) was determined by LF-NMR, which can reflect the fluidity and distribution of water molecules in surimi gels. As shown in Figure 6A, the relaxation components T_21_ (0.1–10 ms), T_22_ (10–300 ms), and T_23_ (>300 ms) represented the bound water, immobilized water, and free water, respectively [31]. And the quantitatively distribution of water state was shown in Figure 6B. Compared to the control group, the T_22_ peak area of the SPI-rich group exhibited a leftward shift and an increase in the peak area, which suggests that the interaction between protein and water has become more stable and showed the highest proportion of immobilized water (T_22_) and the lowest proportion of free water (T_23_). This redistribution of water states confirms the superior water retention capacity of the gel matrix formed by the SPI-rich blend. Indicating the shortest T_2_ relaxation time and the most restricted water mobility. This suggests the formation of a tighter protein network that more effectively confines water molecules. Conversely, a rightward shift was observed for the EWP-rich group, implying a relatively looser network structure with higher water mobility. The YP-rich group exhibited a moderate improvement, while the EWP-rich group showed moisture distribution characteristics similar to the control, aligning well with its water-holding capacity results.

Magnetic resonance imaging provided visual evidence supporting the microstructural differences (Figure 6C). The SPI-rich surimi gels not only appeared smoother but also demonstrated highly uniform color distribution in thermal images, indicating excellent surface thermal homogeneity. This uniformity reflects a dense and coherent internal structure with fewer defects or water channels, facilitating uniform heat transfer. In contrast, the control and EWP-rich surimi gels displayed a slightly mottled thermal distribution, suggesting inferior structural uniformity.

The observed SPI-rich outperformed YP-rich, while EWP-rich performed the worst. This can be attributed to their unique gelation mechanisms and interactions with the myofibrillar protein network. SPI, leveraging its high charge density and capacity for disulfide bond formation [32], synergistically reinforces the network through combined physical filling and chemical cross-linking with myosin [33]. In contrast, while YP promotes myosin cross-linking for a denser structure, its lack of a dominant, strong-gelling component and lower sulfur-containing amino acid content fundamentally limit its ability to form high-strength covalent networks [34]. EWP, with a molecular structure unfavorable for exposing reactive sulfhydryl groups needed for strong disulfide linkages with myosin, acts not as a holistic reinforcement but as a discontinuous filler that compromises the structural integrity and mechanical strength of the composite gel [35].

Furthermore, a slight rightward shift in the T_22_ relaxation time was noted for the high-protein mixtures (Figure 6A). While typically indicative of increased water mobility, this observation in the context of enhanced gel strength and water retention suggests a shift in water-binding patterns. The introduction of exogenous proteins introduces abundant hydrophilic groups that compete with myofibrillar proteins for water, potentially forming hydration layers with altered dynamics [36]. The minor increase in T_22_ likely reflects the redistribution of water into larger, moderately bound pools associated with the added proteins, rather than a net loss of water retention. Critically, the enhanced gel quality stems primarily from the physical confinement within the reinforced, fine microstructure. Rheological data confirm that the SPI-rich group forms a denser, more elastic network. This compact structure physically traps water, significantly inhibiting macroscopic flow and leakage—a fact directly reflected in the marked increase in bound and immobilized water (T_21_ + T_22_) and the decrease in free water (T_23_) (Figure 6B). Consequently, minor changes in local water mobility are overwhelmingly offset by the significantly enhanced capillary and physical confinement effects of the superior gel network, ultimately yielding excellent water retention and texture [37].

### 3.7. Fourier Transform Infrared Spectroscopy (FTIR)

**Figure 7:**
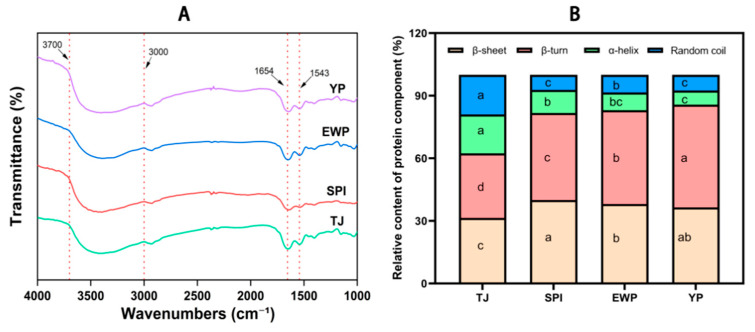
FTIR spectra of surimi gels (**A**) and relative contents of major proteins in surimi gels (**B**); the spectra shown are representative of three independent measurements. Different letters (a–d) indicate significant differences.

FTIR was applied to study the protein secondary structure of surimi gels based on shifts in wavenumber and calculations of peak intensity. The bands of amide I (covering α-helix, β-sheet, β-turn, and random coil structures in the range of 1600–1700 cm^−1^) and amide II (attributed to C–N and N–H stretching vibrations in the range of 1500–1600 cm^−1^) are commonly utilized to analyze the cross-linked protein network in surimi gels, while other absorption bands in the spectra may also offer relevant information [38]. As shown in Figure 7A, all samples exhibited two prominent absorption peaks near 1630 cm^−1^ and 1540 cm^−1^. Which was related to the amide I and the amide II. The overall shapes of these peaks were similar across groups, indicating that the addition of external proteins did not lead to the formation of new covalent bonds. Notably, the SPI-rich and YP-rich groups showed relatively sharper absorption peaks in the amide I region, suggesting a more ordered secondary structure composition. In contrast, the absorption peak for the EWP-rich group was slightly broader, potentially indicating lower structural order or a more complex mixture of conformations.

Supplementary Figure S1 presents the second-derivative spectra of surimi supplemented with different co-formulated protein blends. Furthermore, to quantitatively resolve the secondary structure of proteins, the amide I band was deconvoluted and subjected to curve fitting, providing more detailed structural information from the spectral data. As shown in Figure 7B, the addition of exogenous proteins significantly increased the contents of both β-sheets and β-turns compared with the control group. Among them, the SPI-rich group exhibited the highest β-sheet content, which extended rigid lamellar structures and contributed crucial mechanical strength to the gel by forming dense hydrogen-bond networks [39]. The YP-rich group showed higher β-turn content than the EWP-rich group but lower random coil content, suggesting that the YP-rich gel formed a more ordered protein network.

### 3.8. Analysis of Molecular Interaction Forces

The chemical interaction forces, including ionic bonds, hydrogen bonds, hydrophobic interactions, and disulfide bonds, were quantitatively analyzed in surimi gels. As shown in Table 7, the hydrophobic interactions and disulfide bonds were the main interaction forces in the samples, and the addition of three exogenous proteins enhanced the intermolecular interactions to varying degrees, especially SPI and YP. The hydrophobic interactions were significantly increased from 0.3247 mg/mL to 0.9406 mg/mL in the YP-rich group. This can be attributed to the presence of abundant membrane proteins and structurally complex storage proteins in yeast cells, which are often rich in hydrophobic amino acids. During gel formation, partial denaturation or exposure of hydrophobic regions could occur, and highly efficient hydrophobic aggregation could be generated [40]. The SPI-rich group also exhibited a high hydrophobic interaction content (0.8674 mg/mL). It has been reported that SPI generated self-hydrophobic aggregation and interacted with exposed hydrophobic regions of myofibrillar, forming extensive hydrophobic domains [41]. The slightly lower value for the EWP-rich group (0.5382 mg/mL) may be due to the relatively lower proportion of internal hydrophobic regions in EWP [42].

As for disulfide bonds, the SPI-rich group exhibited the highest content (1.0137 mg/mL), followed by the YP-rich group (1.5729 mg/mL), while no significant difference was observed between the EWP-rich group and the control group. These results can be attributed to the abundant cysteine residues in the 7S and 11S globulin subunits of SPI [43]. During thermal denaturation, the exposed active sulfhydryl groups engaged not only in intramolecular disulfide rearrangement but also in intermolecular disulfide exchange reactions with sulfhydryl groups of myofibrillar proteins, resulting in a highly dense covalent cross-linking network [44]. In contrast, the primary proteins in egg whites (such as ovalbumin) undergo thermal gelation processes that rely more heavily on the construction of strong hydrogen-bond networks. Consequently, they contribute minimally to the overall disulfide bond content of the system [45]. In addition, Zhou [46,47] compared the interfacial properties of SPI and yeast cell wall proteins and found that yeast proteins exhibit unique surface hydrophobicity and charge distribution. This may explain why the YP-rich group strongly promotes hydrophobic interactions similar to the SPI-rich group, despite differences in disulfide bond content.

The addition of exogenous proteins significantly increased the hydrogen bond content in all supplemented surimi gels. This enhancement is attributed to thermal denaturation, which unfolds the proteins and exposes abundant polar side chains, providing additional hydrogen-bonding sites and promoting hydration layer formation with water [48,49]. Both YP and SPI are rich in polar and amide-containing amino acids, which act as strong hydrogen bond donors and acceptors. Upon heating, these proteins further unfold, facilitating the establishment of a dense hydrogen-bond network with fish proteins and water [50,51].

The incorporation of SPI or YP enhanced ionic interactions in surimi gels by introducing abundant charged groups. SPI provides substantial negative charges through its acidic amino acids, which form electrostatic cross-links with positively charged regions of myofibrillar proteins [52,53]. YP, with its broad isoelectric point and amphoteric charge distribution, can simultaneously interact with both positively and negatively charged sites on fish proteins, acting as an effective ionic crosslinker [54,55]. In contrast, EWP has an isoelectric point near pH 4.5, which may locally shift pH away from the optimum for electrostatic attraction. Additionally, thermal denaturation could partially shield its charged groups, limiting ionic bond formation [56,57]. This explains the intermediate ionic bond content observed in the EWP-rich group compared to the control and the SPI-rich or YP-rich groups.

### 3.9. Scanning Electron Microscopy (SEM)

**Figure 8:**
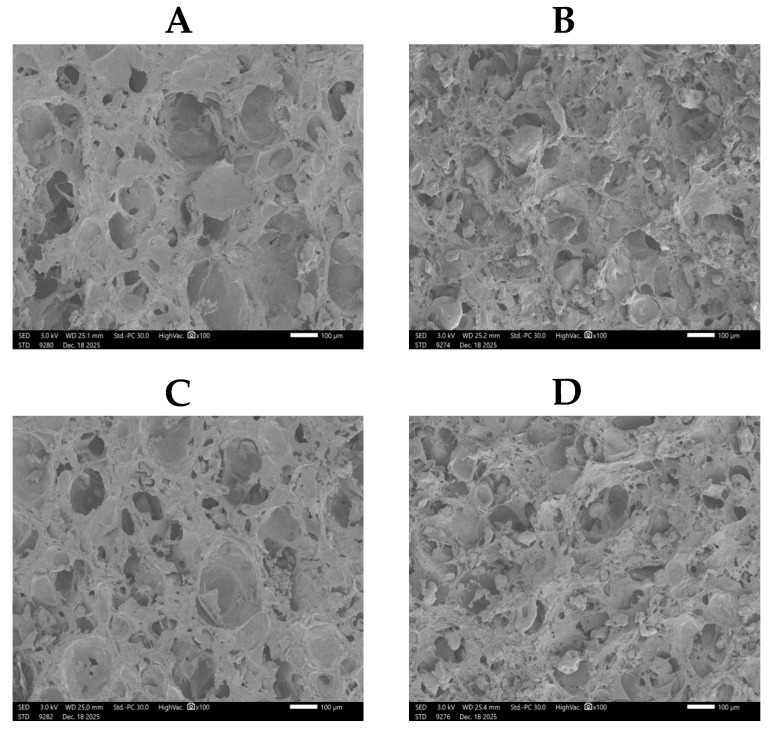
The morphological profiles of surimi gels by SEM; the micrographs shown are representative images obtained from three independent samples. (**A**) TJ group, (**B**) SPI group, (**C**) YP group, (**D**) EWP group.

SEM analysis revealed distinct microstructural characteristics among the surimi gels with different protein additives (Figure 8). The SPI-rich group (B) exhibited a continuous, fine-stranded network with uniform porosity and well-distributed small pores, indicating the formation of a dense and homogeneous protein matrix through effective molecular interactions. In contrast, the EWP-rich group (C) displayed a coarse, discontinuous structure with large, irregular pores and visible structural gaps, reflecting poor integration and weak network development. The YP-rich group (D) showed an intermediate morphology characterized by a heterogeneous yet interconnected porous network, suggesting partial matrix formation with moderate structural integrity. The control gel (A) presented a typical surimi protein network with relatively ordered but less reinforced porosity. These structural observations align closely with the textural, rheological, and hydration properties, confirming that SPI facilitates the most cohesive and stable gel matrix, whereas EWP results in structural discontinuity and functional inferiority.

## 4. Conclusions

In conclusion, this study definitively identifies SPI as the optimal functional additive for enhancing anchovy–threadfin bream composite surimi gel properties. The SPI-rich group facilitated the formation of a dense, continuous protein network through robust hydrophobic interactions and disulfide bonding, resulting in superior gel strength (3551.76 ± 185.83 g), texture, and water retention. YP provided moderate gel reinforcement via hydrophilic entrapment, but its efficacy was limited by inherent structural heterogeneity. In contrast, EWP exhibited inferior performance due to multiple mechanistic factors: electrostatic repulsion from isoelectric point incompatibility (pI mismatch between EWP ≈ 4.5 and fish proteins ≈ 5.0–5.5), reliance on weak hydrogen bonding instead of stronger hydrophobic/disulfide interactions, mismatched gelation kinetics (gelation temperature does not match the surimi), and the formation of a discontinuous microstructure with poor water-holding capacity.

These findings, collectively validated through multi-technique analysis (LF-NMR, SEM, FTIR), emphasized that optimal gel performance depends on the synergistic interaction of multiple factors rather than the effect of a single mechanism. From an industrial perspective, the SPI-dominant formulation offers economic advantages due to the lower cost of soy protein. However, scalability testing under commercial processing conditions and consideration of interactions with other food additives are necessary for practical application. Future work should address limitations such as fixed moisture content and long-term storage stability and employ molecular dynamics simulations for deeper mechanistic insights. This study provides a precise, evidence-based strategy for rational protein selection in developing high-quality surimi products from underutilized anchovy resources, enabling value-added utilization.

## Figures and Tables

**Table 1:** Fish surimi gel basic recipe ingredients list.

Raw Materials	Content
Anchovy	44.29%
Golden thread fish	14.76%
Ice-water	22.14%
Table salt	2.11%
Compound phosphate	0.32%
Cassava starch	3.80%
White sugar	1.05%
Monosodium glutamate	1.37%
TGase	0.16%
Exogenous protein addition	10.00%

**Table 2:** Preparation of composite surimi gels enriched with soy protein isolate, egg white powder, or yeast protein.

Rich Group	SPI	EWP	YP
Soy protein isolate (%)	5.0	2.5	2.5
Yeast protein (%)	2.5	2.5	5.0
Egg white powder (%)	2.5	5.0	2.5

**Table 3:** I-Optimal mixed design experimental operation groups.

Runs	Soy Protein Isolate (%)	Egg White Powder (%)	Yeast Protein (%)
1	6.00	2.00	2.00
2	2.00	6.00	2.00
3	2.00	2.00	6.00
4	4.00	4.00	2.00
5	4.00	2.00	4.00
6	2.00	4.00	4.00
7	4.67	2.67	2.67
8	2.67	4.67	2.67
9	2.67	2.67	4.67
10	3.33	3.33	3.33

**Table 4:** ANOVA component analysis table for the optimized formula of surimi gels.

Attributes	Predicted Model	*p*-Value	R^2^	Lack of Fit (*p*-Value)	R^2^ (Adjusted)
Elasticity	298.26 × A + 179.13 × B + 274.81 × C − 808.01 × AB − 987.96 × AC − 745.31 × BC + 1196.93 × ABC − 108.00 × A^2^BC + 1897.20 × AB^2^C	0.0054	0.9763	0.3112	0.9588
Toughness	264.08 × A + 136.55 × B + 164.83 × C − 648.56 × AB − 700.119 × AC − 440.66 × BC + 970.69 × ABC − 712.80 × A^2^BC + 1465.20 × AB^2^C	0.0006	0.9925	0.2028	0.9774
Smoothness	151.83 × A + 80.51 × B + 104.01 × C − 306.37 × AB − 345.97 × AC − 204.52 × BC + 552.56 × ABC − 421.20 × A^2^BC + 518.40 × AB^2^C	0.0068	0.9731	0.1337	0.9694
Total	460.19 × A + 242.37 × B + 376.34 × C − 1089.37 × AB − 1351.04 × AC − 894.07 × BC + 1501.52 × ABC − 467.99 × A^2^BC + 2654.39 × AB^2^C	0.0229	0.9497	0.1633	0.9271

**Table 5:** Polynomial regression equations for each indicator.

Attributes	Elasticity	Toughness	Smoothness	Total
A × B	0.0018	0.0005	0.0028	0.0143
A × C	0.0008	0.0004	0.0018	0.0068
B × C	0.0024	0.0022	0.0118	0.0271

**Table 6:** Textural profile analysis (TPA) attributes of surimi gels with different dominant proteins. Different letters (a–d) indicate significant differences.

	Hardness (g)	Chewiness (g)	Gumminess (g)	Springiness	Resilience	Cohesiveness (g·s)
TJ	2594 ± 334 ^c^	1541 ± 361 ^c^	1333 ± 336 ^c^	0.86 ± 0.02 ^b^	0.59 ± 0.07 ^c^	0.33 ± 0.05 ^c^
SPI	3552 ± 186 ^a^	2724 ± 253 ^a^	2401 ± 223 ^a^	0.88 ± 0.01 ^a^	0.77 ± 0.04 ^a^	0.45 ± 0.02 ^a^
EWP	1793 ± 204 ^d^	632 ± 138 ^d^	516 ± 91 ^d^	0.82 ± 0.04 ^c^	0.35 ± 0.04 ^d^	0.15 ± 0.03 ^d^
YP	2948 ± 235 ^b^	1918 ± 217 ^b^	1655 ± 200 ^b^	0.86 ± 0.02 ^b^	0.65 ± 0.04 ^b^	0.37 ± 0.02 ^b^

**Table 7:** Molecular interactions involved in surimi gels. Different letters (a–d) indicate significant differences.

	Ionic Bonds (mg/mL)	Hydrogen Bonds (mg/mL)	Hydrophobic Interactions (mg/mL)	Disulfide Bonds (mg/mL)
TJ	0.1097 ± 0.0364 ^c^	0.1150 ± 0.0175 ^d^	0.3247 ± 0.1343 ^d^	1.0137 ± 0.2645 ^c^
SPI	0.1777 ± 0.0316 ^a^	0.3292 ± 0.0395 ^c^	0.8674 ± 0.1997 ^b^	1.8289 ± 0.3507 ^a^
EWP	0.1225 ± 0.0236 ^b^	0.3449 ± 0.0552 ^b^	0.5382 ± 0.1295 ^c^	1.0974 ± 0.2808 ^c^
YP	0.1755 ± 0.0294 ^ab^	0.3696 ± 0.0369 ^a^	0.9406 ± 0.2729 ^a^	1.5729 ± 0.1947 ^b^

## Data Availability

The original contributions presented in the study are included in the article/Supplementary Material, further inquiries can be directed to the corresponding authors.

## Supplementary Materials

The following supporting information can be downloaded at: https://www.mdpi.com/article/10.3390/foods15081417/s1, Figure S1: Fourier Infrared Second Derivative Image.
