# Supplementary figures and images for: Optimization of Anchovy–Threadfin Bream Composite Surimi: I-Optimal Mixture Design for Sensory Enhancement and Impact Assessment of Three Exogenous Proteins
Source: Foods. 2026 Apr 17;15(8):1417. doi: 10.3390/foods15081417 (PMC13115514; doi:10.3390/foods15081417)

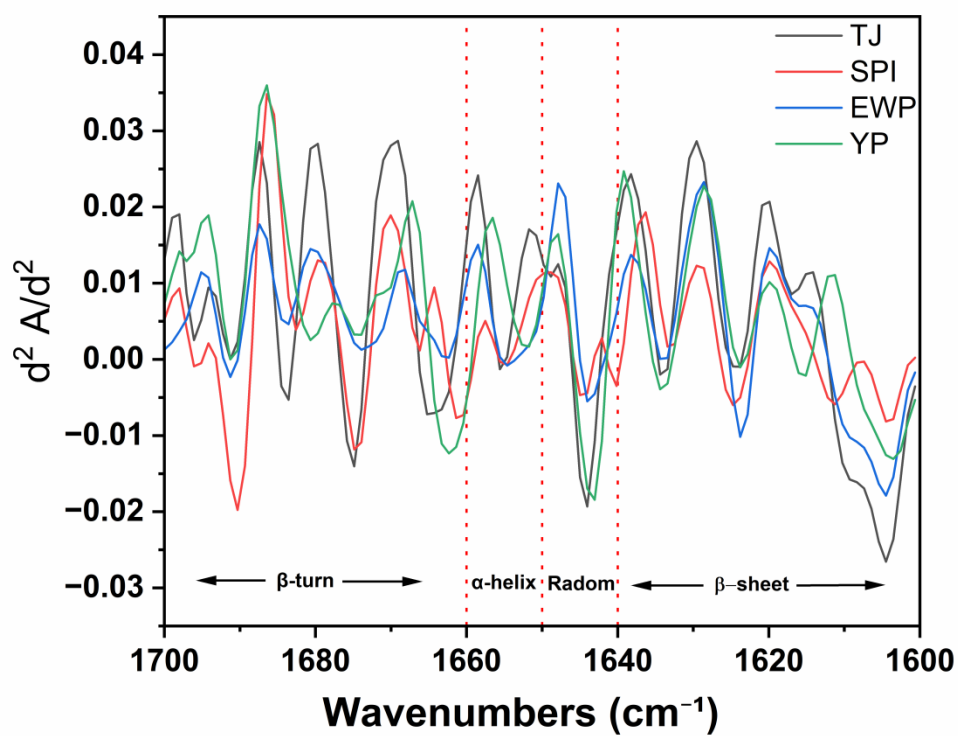

**Figure S1.** Fourier Infrared Second Derivative Image

Supplement: Supplementary file 1 [file foods-15-01417-s001.zip › foods-4218462-supplementary.pdf]
